# Parenting and Health: The Major Challenge of Complex Relations

**DOI:** 10.3390/children8100928

**Published:** 2021-10-17

**Authors:** Dora Pereira

**Affiliations:** 1 Department of Psychology, Faculty of Arts and Humanities, University of Madeira, 9020-105 Funchal, Portugal; dora.pereira@staff.uma.pt; 2 Research Centre for Regional and Local Studies, University of Madeira, 9020-105 Funchal, Portugal

Parenting is an interpersonal process associated with taking care and fostering the healthy development of children and young people. Parental behavior stems from the characteristics of three functional components: parenting capacity, mediating processes, and parenting skills [1]. It implies the mobilization of emotional, cognitive, and behavioral resources of parents, resulting from the interaction between the characteristics of their psychic structure; the context in which they live and their relational network (parenting capacity); and when it is recognized, the relevance (mediating processes) of developing parenting behaviors (parenting skills) that aim to ensure the effective and timely satisfaction of child’s specific needs. That is, parental capacity is operationalized in parenting skills when the relevance of such actions is recognized. Thus, parenting requires enough resources to learn or carry out the behaviors that appear to be more appropriate and necessary to foster the child’s good development, and capacity to recognize what these behaviors are and how should they be updated throughout the child’s life according to their developmental characteristics. As an example, the setting of rules and limits must change to consider the child’s cognitive and emotional developmental features and promote his or her social inclusion. As Barudy and Dantagnan [2] point out, taking good care of a child stems from the balance between the child’s needs and parenting skills, in interaction with the characteristics of the context, namely how it supports parenting and facilitates access to enough and adequate resources (e.g., childcare programs or specialized interventions), and the parents’ resilience processes in the face of adversity. Parenting implies—universally—the promotion of the child’s health, but also to cope with specific risk factors and, in some situations or periods of life, to deal with conditions that harm health and may prevent the mobilization of necessary resources for parenting (e.g., parents’ mental health problems), or require specific parenting skills (e.g., children’s metabolic diseases). If a child’s needs require skills that exceed the parents’ resources to acquire or mobilize them in due course, the risk of abuse or neglect increases. The impact of these risk factors or conditions can increase the likelihood of parental neglect or maltreatment of children and jeopardize their health in the short, medium, and long term. Adverse childhood events include abusive experiences and are recognized as an important factor impacting adults’ health [3]. From this perspective, more or less adequate parental behavior refers (also) to how the complex parenting system [4], in which multiple variables interact uninterruptedly and simultaneously—ensures the health of its members.

Health is a state of physical, psychological, and social well-being [5], but also a major resource for daily life, an experience, an ability, or a phenomenon [6]. Considering health as a goal (to achieve or maintain) led to approaches towards its promotion (primary prevention), the decrease of the impact of risk factors or the prevention of the evolution of early stages of a disease (secondary prevention) and dealing with existing conditions in order to recover a general state of health and prevent future relapses (tertiary prevention), prevent over diagnosis and treatment (quaternary prevention), and prevent misinformation and its negative effects on the health of individuals (quinary prevention) [7]. Parenting is considered a determinant of health, influencing several key processes as neurodevelopment, attachment, or stress response [8] in high or low risk contexts [9]. However, parenting is also influenced by health conditions, and both could be conceived as an INUS condition of the other: good parenting (or good health) is insufficient to bring good health (or good parenting) but is a necessary part of an unnecessary set of conditions [10]. This bidirectional relationship between parenting and health can be researched from various perspectives, among which three stand out and frame the papers included in this Special Issue. First, research focused on how parenting influences a child’s health, which includes the study of the contribution of specific characteristics of parental behavior for the health of children, such as the presence and involvement of parents in childcare [11], or their attitude when shopping for toys [12]. Second, research aiming to understand how conditions that impact (or may come to) the health of parents and children influence parenting capacity or mediating processes. This line includes the papers of Burns et al. [13], about the practice of physical activity; Uhm and Kim [14] focused on the impact of diabetes on parents’ quality of life; and Weaver et al. [15] centered on parenting in the face of complex medical needs. Third, parental behavior in the face of interventions (medical, psychological, social, educational, or multidisciplinary) raised by health needs, developed in different contexts (school, hospital, domestic, residential care, etc.) that may or may not require the learning of new parenting skills has been studied, which includes the work of Palomo-Carrión [16].

Throughout the life cycle, parenting and health are deeply intertwined; the articles present in this Special Issue intend to contribute to better understand this complex relation.

## References

[1] Pereira D., Nunes L.M., Fonte C., Alves S.P., Sani A.I., Estrada R., Caridade S. (2019). Parentalidade. Comportamento e Saúde Mental: Dicionário Enciclopédico.

[2] Barudy J., Dantagnan M. (2005). Los Buenos Tratos a la Infancia: Parentalidade, Apego y Resiliência.

[3] Symonds L.J. (2020). Childhood trauma: The cause that needs a cure. Life Res..

[4] Stevens I., Cox P. (2008). Complexity Theory: Developing New Understandings of Child Protection in Field Settings and in Residential Child Care. Br. J. Soc. Work.

[5] World Health Organization (2020). Basic Documents: Forty-Ninth Edition (Including Amendments Adopted up to 31 May 2019).

[6] McCartney G., Popham F., McMaster R., Cumbers A. (2019). Defining health and health inequalities. Public Health.

[7] Dutta D., Arora V., Dhingra A., Das A.K., Fariduddin M., Shaikh K., Priya G. (2021). Quinary Prevention in Diabetes Care: Need for Multidisciplinary Approach. Clin. Epidemiol. Glob. Health.

[8] Shah N., Stewart-Brown S. (2018). Parenting and health: A call for action. Paed Child. Healt.

[9] Stern J.A., Beijers R., Ehrlich K.B., Cassidy J., de Weerth C. (2020). Beyond Early Adversity: The Role of Parenting in Infant Physical Health. J. Dev. Behav Pediatr.

[10] Meehl P.E. (2001). Comorbidity and taxometrics. Clin. Psychol.-Sci. Pr..

[11] Pablo L.A., Erkhembayar R., Davison C.M. (2021). Father Presence, Father Engagement, and Specific Health and Developmental Outcomes of Mongolian Pre-School Children. Children.

[12] Lipowska K., Łada-Maśko A.B. (2021). When Parents Go Shopping: Perspectives on Gender-Typed Toys among Polish Mothers and Fathers from Big Cities. Children.

[13] Burns R.D., Colotti T.E., Pfledderer C.D., Fu Y., Bai Y., Byun W. (2020). Familial Factors Associating with Youth Physical Activity Using a National Sample. Children.

[14] Uhm J.-Y., Kim M.S. (2020). Predicting Quality of Life among Mothers in an Online Health Community for Children with Type 1 Diabetes. Children.

[15] Weaver M.S., Neumann M.L., Lord B., Wiener L., Lee J., Hinds P.S. (2020). Honoring the Good Parent Intentions of Courageous Parents: A Thematic Summary from a US-Based National Survey. Children.

[16] Palomo-Carrión R., Romay-Barrero H., Romero-Galisteo R.-P., Pinero-Pinto E., López-Muñoz P., Martínez-Galán I. (2020). Modified Constraint-Induced Movement Therapy at Home—Is It Possible? Families and Children’s Experience. Children.

